# Supplementary figures and images for: Quantitative magnetic resonance imaging predicts individual future liver performance after liver resection for cancer
Source: PLoS One. 2020 Dec 2;15(12):e0238568. doi: 10.1371/journal.pone.0238568 (PMC7710097; doi:10.1371/journal.pone.0238568)

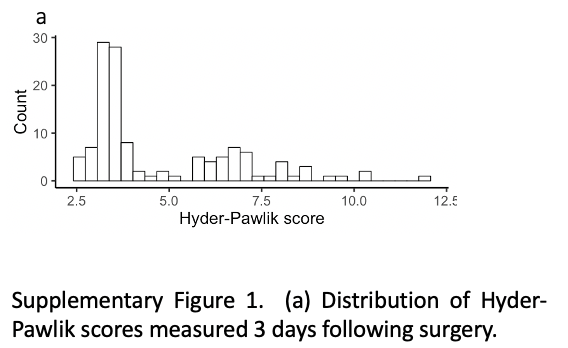

Supplement: S1 Fig — (TIF) [file pone.0238568.s001.tif]
