# Supplementary material for: Quantitative magnetic resonance imaging predicts individual future liver performance after liver resection for cancer
Source: PLoS One. 2020 Dec 2;15(12):e0238568. doi: 10.1371/journal.pone.0238568 (PMC7710097; doi:10.1371/journal.pone.0238568)
Supplement: Attachment — Submitted filename: PLOS Review - Quantitative magnetic resonance imaging predicts individual future liver performance after liver resection for cancer.docx [file pone.0238568.s002.docx]

The authors uses quantitative MRI to determine pre-operative liver health and combined this with future liver remnant measurements to predict future liver performance after liver resection. The results showed that a combination of low future liver remnant volume and elevated corrected T1 liver measurements were able to predict poor future liver performance. While the work shows significant potential there are several critiques and unanswered questions

1. The authors do not define what criteria was used to determine poor post-operative liver performance. Please define this criteria and show the comparison between the high risk group and the low risk group for predictive post-operative poor liver function.
2. Length of stay of 1.5 days long in the high risk group compared to the low risk group. Were other factors that may have contributed to this, such as age, comorbidities, extent of resection, etc., controlled for?
3. The AUROC and confidence intervals for the predictive value of FLR and the hepatica score strongly overlap. I am not sure it can definitively be said the hepatica score is a better predictor. If anything it adds only a modest increase in predictive value.
4. Only 21% of the patients had abnormal liver parenchyma and none had histopathologic proven cirrhosis. These are the patients that a predictive score would be most applicable to. It would be ideal to see a larger percentage of patients with abnormal liver parenchyma and to include patients with cirrhosis in order to draw stronger conclusions that would be more broadly applicable.
