# Supplementary material for: Quantitative magnetic resonance imaging predicts individual future liver performance after liver resection for cancer
Source: PLoS One. 2020 Dec 2;15(12):e0238568. doi: 10.1371/journal.pone.0238568 (PMC7710097; doi:10.1371/journal.pone.0238568)
Supplement: Attachment — Submitted filename: PLOS_One response to reviewers_v2.docx [file pone.0238568.s003.docx]

Dr John Connell

Senior Scientist

Perspectum Ltd

Gemini One, 5520 John Smith Drive

Oxford, OX4 2LL

United Kingdom

+44(0) 1865 655343

24^th^ July2020

To Dr Leonidas G Koniaris

Dear Editor,

Thank you for your editorial review of our manuscript entitled **Quantitative magnetic resonance imaging predicts individual future liver performance after liver resection for cancer**. We enclose a response to the reviewer’s comments as well as addressing additional editorial requirements. We thank the reviewer for their comments, which improve the paper, and we have added a comment in the Acknowledgements section for this

**Comments to the author and responses:**

*Reviewer #1: The authors uses quantitative MRI to determine pre-operative liver health and combined this with future liver remnant measurements to predict future liver performance after liver resection. The results showed that a combination of low future liver remnant volume and elevated corrected T1 liver measurements were able to predict poor future liver performance. While the work shows significant potential there are several critiques and unanswered questions*

1. ***The authors do not define what criteria was used to determine poor post-operative liver performance. Please define this criteria and show the comparison between the high risk group and the low risk group for predictive post-operative poor liver function.***

The criterion used is the modified Hyder-Pawlik score, as defined on page 7 of the manuscript. We have inserted text into the manuscript (legend text of Figure 5a, page 11) to clarify this further including the weightings previously applied by Hyder&Pawlik.

We thank the Reviewer for the recommendation to show the comparison between the high-risk group and the low-risk group for predicting post-operative poor liver function. We have inserted this into Figure 5b and Figure 5 is now re-uploaded.

2. ***Length of stay of 1.5 days long in the high risk group compared to the low risk group. Were other factors that may have contributed to this, such as age, comorbidities, extent of resection, etc., controlled for?***

It is correct that there are other factors that might be conceived to affect length of stay and we have examined the following: presence of comorbidities, resection volume greater than 50%, preoperative bilirubin level above upper limit of normal (20 µmol/L), total lesion volume above 100ml, age over 60 years, BMI over 25 kg/m^2^, liver fat over 10%. However, none of these showed no significant difference (P>0.05) in the length of stay. Please see graphs and associated p-values below. We have added a comment relating to this in the manuscript (page 10). Additionally, the distribution of each of these covariates is tabulated below indicating that FLR, bilirubin, age and lesion volume are not significantly different between the cT1 risk groups.

As previously published on extensive data analysis from over 2000 patients in the UKBiobank (Mojtahed et al., 2019 *Abdominal Radiology*), a higher cT1 was associated with an elevated liver PDFF and an elevated BMI. Those conclusions are consistent with the results from the cohort of patients in this study. However, BMI and PDFF were not independent predictors of length of stay.

3. ***The AUROC and confidence intervals for the predictive value of FLR and the hepatica score strongly overlap. I am not sure it can definitively be said the hepatica score is a better predictor. If anything it adds only a modest increase in predictive value.***

We thank the reviewer for the assessment of the two AURCOC and confidence intervals for the FLR and Hepatica score. We accept that the improvement in predictive capacity is modest, and we contend that this is reflected in the language that we have used to describe the strength of this conclusion we have drawn in the manuscript.

Nevertheless, a statistical examination of this difference using the DeLong’s test for two correlated ROC curves indicates that the difference between the two predictive models is statistically significant (P-value = 0.000104). We have now added this into the manuscript (see page 11, yet we have maintained our existing conclusions.

4. ***Only 21% of the patients had abnormal liver parenchyma and none had histopathologic proven cirrhosis. These are the patients that a predictive score would be most applicable to. It would be ideal to see a larger percentage of patients with abnormal liver parenchyma and to include patients with cirrhosis in order to draw stronger conclusions that would be more broadly applicable.***

Existing guidelines from AASLD, EASL and APASL are in agreement that patients presenting with very early and early stage HCC (Barcelona Clinic Liver Cancer stage 0/A) achieve the best outcomes when surgery is offered where the Child-Pugh score indicates no cirrhosis. For secondary liver cancer, metastases often appear on the backdrop of an otherwise healthy liver parenchyma.

The experienced surgeons leading the study (Mole, Sherif, Welsh, Rees) consider that the cases where liver fibroinflammation has not previously been diagnosed presents the greatest risk to the development of post-operative liver failure following surgical resection. As such, we anticipate this new technology to be applied pre-operatively to all-comers being considered for liver resection such that biopsy confirmation cirrhosis is not required, therefore avoiding well document biopsy-associated risks such as bleeding.

Nevertheless, we concur with the proposition that “It would be ideal to see a larger percentage of patients with abnormal liver parenchyma.” We are already undertaking subsequent examinations of this technology with participants that we expect to display a greater level of parenchymal liver disease (see: <https://clinicaltrials.gov/ct2/show/NCT04451603>). We eagerly await the results of this study and will reference the publication of this manuscript.

I hope that our responses, revisions to the manuscript and new analyses are acceptable to the reviewer.

**Response to additional journal requirements:**

1. The formatting has been edited to meet PLOS ONE’s style requirements.
2. The table has been included as part of the main manuscript, and Table1 has been removed as a separate file.

All relevant data are within the manuscript and its Supporting Information Files. This is now stated in the Data Availability statement. The data underlying the results presented in the study are available from <https://datashare.is.ed.ac.uk/handle/10283/3716>

a. Our Funding Statement is now the following:

'We gratefully acknowledge the funders of this work through an Early Stage Biomedical Catalyst Award from Innovate UK, part of UK Research and Innovation, ref: 79891-510341. DJM is funded by the UK Medical Research Council through a Senior Clinical Fellowship, ref: MR/P008887/1. JAF was funded through an NHS Research Scotland/Universities Senior Clinical Fellowship. Additional funding was contributed by North Hampshire Medical Fund. The funder provided support in the form of salaries for authors, but did not have any additional role in the study design, data collection and analysis, decision to publish, or preparation of the manuscript. The specific roles of these authors are articulated in the ‘author contributions’ section.”MK, GR, JC, JMcG, RB, MB are employed by Perspectum'

b. The Competing Interests Statement is now amended.

4. The HepaT1ca study group has been revised to include affiliations and a contact email address is provided.
5. Supporting information caption is now added at the end of the manuscript.

Thank you for your time and consideration of our manuscript, we look forward to hearing from you.

Yours sincerely,

Dr John Connell
